# Complete Chloroplast Genomes of *Erianthus arundinaceus* and *Miscanthus sinensis*: Comparative Genomics and Evolution of the *Saccharum* Complex

**DOI:** 10.1371/journal.pone.0169992

**Published:** 2017-01-26

**Authors:** Shin-ichi Tsuruta, Masumi Ebina, Makoto Kobayashi, Wataru Takahashi

**Affiliations:** 1 Japan International Research Center of Agricultural Sciences, Tropical Agriculture Research Front (JIRCAS-TARF), Okinawa, Japan; 2 National Agriculture and Food Research Organization, Institute of Livestock and Grassland Science (NARO-ILGS), Tochigi, Japan; Austrian Federal Research Centre for Forests BFW, AUSTRIA

## Abstract

The genera *Erianthus* and *Miscanthus*, both members of the *Saccharum* complex, are of interest as potential resources for sugarcane improvement and as bioenergy crops. Recent studies have mainly focused on the conservation and use of wild accessions of these genera as breeding materials. However, the sequence data are limited, which hampers the studies of phylogenetic relationships, population structure, and evolution of these grasses. Here, we determined the complete chloroplast genome sequences of *Erianthus arundinaceus* and *Miscanthus sinensis* by using 454 GS FLX pyrosequencing and Sanger sequencing. Alignment of the *E*. *arundinaceus* and *M*. *sinensis* chloroplast genome sequences with the known sequence of *Saccharum officinarum* demonstrated a high degree of conservation in gene content and order. Using the data sets of 76 chloroplast protein-coding genes, we performed phylogenetic analysis in 40 taxa including *E*. *arundinaceus* and *M*. *sinensis*. Our results show that *S*. *officinarum* is more closely related to *M*. *sinensis* than to *E*. *arundinaceus*. We estimated that *E*. *arundinaceus* diverged from the subtribe Sorghinae before the divergence of *Sorghum bicolor* and the common ancestor of *S*. *officinarum* and *M*. *sinensis*. This is the first report of the phylogenetic and evolutionary relationships inferred from maternally inherited variation in the *Saccharum* complex. Our study provides an important framework for understanding the phylogenetic relatedness of the economically important genera *Erianthus*, *Miscanthus*, and *Saccharum*.

## Introduction

The Poaceae is the grass family comprised of approximately 700 genera and more than 10,000 species and grouped into two major clades, BEP (the subfamilies Bambusoideae, Ehrhartoideae, and Pooideae) and PACMAD (the subfamilies Panicoideae, Arundinoideae, Chloridoideae, Micrairoideae, Aristidoideae, and Danthonioideae) [1–3]. The Andropogoneae is one of the tribes of the Panicoideae that includes many economically important C_4_ grasses such as maize (*Zea mays* L.), sorghum (*Sorghum bicolor* L. Moench), and sugarcane (*Saccharum* spp.). The genera *Saccharum*, *Erianthus*, and *Miscanthus* are members of the subtribe Saccharinae within the Andropogoneae [4]. *Erianthus* and *Miscanthus* exhibit diverse important agricultural traits such as high productivity, high percentage of dry matter, good ratooning ability, vigor, and resistance to environmental stresses [5–7]. These genera are cross-compatible with *Saccharum* species [8], and sugarcane breeders have created intergeneric hybrids between commercial *Saccharum* spp. hybrids and these genera [9–12]. Thus, *Erianthus* and *Miscanthus* have attracted attention as potential genetic resources for sugarcane improvement [13–15]. In addition to their favorable agricultural traits, the low ash content and high heating value make *Erianthus* and *Miscanthus* promising cellulosic feedstocks at energy conversion plants; they can be used for methanol synthesis by gasification and for direct combustion [6, 7, 16]. Ongoing studies focus on the conservation and use of wild *Erianthus* and *Miscanthus* accessions as breeding materials [17, 18].

Despite current interest, the taxonomy and phylogenetic relatedness of *Saccharum* and these related genera have been controversial until recently, because the common criterion, variation of the awn on the lemma, used for differentiation within these genera does not clearly distinguish between the genera [12]. Therefore, *Erianthus* and *Miscanthus* have been regarded by some taxonomists as being synonymous with *Saccharum* and have been grouped into the so-called ‘*Saccharum* complex’ [19], which includes the members of *Saccharum* L., *Erianthus* Michx., *Miscanthus* Anderss., *Narenga* Bor, *Sclerostachya* A. Camus. This theory is widely accepted by sugarcane breeders [20].

Phylogenetic analyses based on molecular data have been employed to reconstruct the phylogeny of the *Saccharum* complex. In these studies, DNA variation detected by using DNA markers developed from nuclear genomes [8, 10, 14, 17, 21–24], was used to assess genetic diversity among wild accessions in these genera. Welker *et al*. [25] showed that a phylogenetic tree inferred from low-copy nuclear loci was useful for understanding the relationships between polyploid taxa and identifying allopolyploidization events in *Saccharum* and related genera. In addition, the data sets of partial sequences [21] and DNA markers [8, 14, 26–28] developed from organelle genomes were also used to estimate the phylogenetic relationships between the species and genera of the *Saccharum* complex. These studies have provided valuable insight into the phylogenetic relations within the *Saccharum* complex: (1) *Saccharum* is more closely related to *Miscanthus* than to *Erianthus*; (2) *Erianthus* is more closely related to *Sorghum* than to the other members of the *Saccharum* complex; (3) the evolutionary history of *Erianthus* may differ from that of other members of the *Saccharum* complex. These results have indicated the potential of this approach to elucidate the phylogenetic relationships within the *Saccharum* complex.

Because the chloroplast (cp) genome has conserved gene content and uniparental inheritance [29], polymorphism within the chloroplast genome is a valuable tool for phylogenetic and evolutionary studies [30]. To date, only 12 cpDNA markers [14] and 28 partial sequences are registered for *Erianthus arundinaceus* in GenBank; therefore, there is a clear need for additional sequence information on the *E*. *arundinaceus* cp genome. Comparison of the complete cp genome sequences could reveal novel genome features such as single-nucleotide polymorphisms (SNPs), insertions/deletions (indels), and microsatellites. This information would improve the analyses of the relationships in the *Saccharum* complex, especially for *Erianthus*. Multiple alignments of complete cp genomes reveal sequence variability, which is needed for the development of DNA markers for taxonomic and evolutionary studies. In the *Saccharum* complex, the complete cp genome sequences were first reported for *Saccharum officinarum* in 2004 [31, 32] and more recently for *Miscanthus sinensis* [33]. However, as the *E*. *arundinaceus* cp genome has not been fully sequenced, the whole-genome comparison between these major genera of the *Saccharum* complex has not yet been possible.

Recent advances in pyrosequencing, which allows high-throughput sequence analysis of a wide range of genomes, has simplified sequencing, considerably increased its speed, and reduced the cost. This approach enables faster and more efficient determination of whole cp genome sequences, and has been applied to many plant species, including those in the Poaceae [33, 34]. In this study, we present the complete cp genome sequence of *Erianthus arundinaceus* determined using pyrosequencing. On the basis of this sequence, we designed a primer set that is useful for validation of ambiguous sites such as homopolymeric and gap regions in Poaceae cp genomes, and also for sequencing of the entire cp genomes; we used these primers to sequence the whole cp genome of *Miscanthus sinensis*. Our analysis of these cp genomes provides detailed data on the distribution of SNPs, indels, and microsatellites in *Saccharum* and related genera. We also discuss the evolution of the *Saccharum* complex based on the sequence variations of these cp genomes.

## Results

### Assembly and annotation of the chloroplast genomes of *Erianthus arundinaceus* and *Miscanthus sinensis*

The *E*. *arundinaceus* cp genome was sequenced using pyrosequencing on the 454 GS FLX system. A total of 481,406 sequence reads (average, 336 bp; range, 30–897 bp) were generated, representing a 162-Mbp sequence. After filtering the reads by local BLASTN analysis with the *S*. *officinarum* cp genome (GenBank accession No. NC006084) as a reference, 5,052 reads (average, 362 bp) were retained; a 12-fold coverage of the cp genome was reached. There were 30 homopolymeric stretches (≥10 bp), which may lead to errors in the assembled sequences [35]. The accuracy of these regions and the inverted repeat (IR) junction regions in assembled sequences was confirmed by using PCR-based sequencing. Thus, the complete *E*. *arundinaceus* cp genome sequence was obtained. To determine the complete sequence of the *M*. *sinensis* cp genome, we used the Sanger sequencing with primers designed from the *E*. *arundinaceus* cp genome sequence. Sixteen overlapping regions were amplified with specific primers (Table 1) and a total of 320 sequence reads were obtained by using 258 primers, among which 253 primers (98.1%) were identical to both *M*. *sinensis* and *S*. *officinarum* cp genome sequences and 251 (97.3%) were also identical to that of *Sorghum bicolor* (S1 Table).

**Table 1:** Primer pairs used for amplification of *Miscanthus sinensis* cp genome.

Primer pair	Primer sequence (5′ to 3′)		T_m_	PCR product			Length (bp)
	Forward	Reverse	(°C)	Location^1^	Start^2^	End^2^	
ES01	TTGTGAGCATTACGTTCGTGC	GCTGAGTGGTTGATAGCTCCG	60	LSC	140	12113	11974
ES02	TGATCGTGATTTGGAACCTGTTC	ATTGAAGCATCTCGCACCTT	58	LSC	11835	19267	7433
ES03	AATGAAAGGGTCTGGTTGGA	CCAATTGCATGCGTCTAATC	58	LSC	18929	26001	7073
ES04	AGAGTGCCTAATCACGAGGATCC	CCTCTTGTATCATCAACCCATCG	60	LSC	25073	37410	12338
ES05	AACAAAGGGCGATGAATCAG	AACCGTTCAAGCTGTTCCTG	56	LSC	36910	38656	1747
ES06	GTCGAATTTGCAGAAGGGACGAG	GAGTTCTTGTCGCACTCCTTTGTG	60	LSC	37314	50226	12913
ES07	GTGGATTAATCGGACGAGGA	ACTGCAGCTCCTGCTTCTTC	58	LSC	49983	57728	7746
ES08	GCAGGCGCAGATCTATGAAT	CCTTTGCTCTGATGGTTGGAATC	58	LSC	56347	63939	7593
ES09	GGCTAGTTGAGTAGTTTTGATTAAGG	AGACCGTGGAGGATCCACAATAG	60	LSC	63690	76120	12431
ES10	CCATGAACAGGCTCCGTAAG	CGTTATGATACTGAATCTCATGCC	58	LSC	75648	82881	7234
ES11	TGGATTATGACGTGGATTGTATCG	GTAGGACTGGTGCCGACAGTTCATC	58	LSC-IRA	82318	94864	12547
ES12	CCAAACATATGCGGATCAAATCACG	GAATATTGGAGTTAACCATATTATC	56	IRA-SSC	94130	106419	12290
ES13	CCAAATTCCAGATTCCAGCA	AAACCATTGCTTCGTCTGGT	54	SSC	105400	112986	7587
ES14	CCCATGTGAGATACGGAGGA	TGAAATTCTCGAGCCCAAAG	56	SSC	111573	119368	7796
ES15	TGTAAATACCCTAATATAGGTTCGC	CCAAACATATGCGGATCAAATCACG	56	SSC-IRB	118477	130428	11952
ES16	GTAGGACTGGTGCCGACAGTTCATC	TAGGTATTAGTACTATGGCATTC	60	IRB-LSC	129694	290	12013

^1^ LSC: Large single-copy, SSC: Small single-copy, IRA: Inverted repeat A, IRB: Inverted repeat B.

^2^ Position (base pairs) in the *M*. *sinensis* chloroplast genome sequence.

**Fig 1:**
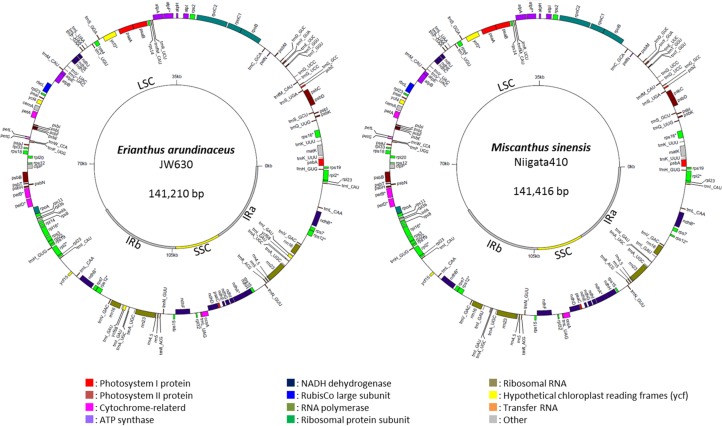
Chloroplast genome maps of *Erianthus arundinaceus* and *Miscanthus sinensis*. The genes of different functional groups are indicated in different colors. Genes on the inside and outside of the maps are transcribed clockwise and counter-clockwise, respectively. The thick lines on the inner circles indicate inverted repeats (IRa and IRb), which separate the genomes into the small single-copy (SSC) and large single-copy (LSC) regions.

The complete cp genomes of *E*. *arundinaceus* (141,210 bp) and *M*. *sinensis* (141,416 bp) had typical circular structures (Fig 1). The cp genome of *E*. *arundinaceus* included a large single-copy (LSC) region (83,170 bp) and a small single-copy (SSC) region (12,516 bp), which were separated by a pair of IRs (IRa and IRb; 22,762 bp each); that of *M*. *sinensis* consisted of an LSC (83,141 bp), an SSC (12,681bp), and two IRs (22,797 bp each). The GC content was 38.5% in the *E*. *arundinaceus* genome and 38.4% in the *M*. *sinensis* genome; these values were similar to those of other Panicoideae including *S*. *officinarum* [31], *M*. *sinensis* [33], and *S*. *bicolor* [36]. The number of genes was 143 in *E*. *arundinaceus* and 141 in *M*. *sinensis*, including 86 and 84 protein–coding genes, respectively. Each genome contained 8 ribosomal RNA (rRNA) genes and 49 transfer RNA (tRNA) genes. Coding genes accounted for 58.9% (*E*. *arundinaceus*) and 58.4% (*M*. *sinensis*) of the genomes (Table 2). The difference in the gene number was due to a difference in *ycf*68 in the IR regions, which appeared to be a pseudogene in *M*. *sinensis* because of a frame-shift mutation. *S*. *officinarum* and *E*. *arundinaceus* have the complete *ycf*68 open reading frame, whereas *S*. *bicolor* has a frame-shift mutation at the same position as in *M*. *sinensis*. The members of the *Saccharum* complex also have lost *acc*D, *ycf*1, and *ycf*2, which are absent in the cp genomes of other Panicoideae grasses [33, 36, 37]. We also found that the start codons of the *rpl*2 and *rps*19 genes are likely to convert to ACG and GTG via RNA editing during translation both in *E*. *arundinaceus* and *M*. *sinensis*, as reported in other species [37–39].

**Table 2:** Characteristics of the chloroplast genomes in three genera of the *Saccharum* complex and *Sorghum bicolor*.

Species	Genome organization^1^					Number of genes^2^				GenBank accession No.
	Length of total genome	Length of LSC	Length of SSC	Length of IR	GC/AT contents (%)	Total	CDS^3^	rRNA	tRNA	
*S*. *officinarum*	141,182	83,048	12,544	22,795	38.4/61.6	136	87 (8) ^4^	8 (4) ^4^	41 (8) ^4^	NC006084 [31]
*E*. *arundinaceus*	141,210	83,170	12,516	22,762	38.5/61.5	136	87 (8)	8 (4)	41 (8)	LC160130 [This study]
*M*. *sinensis*	141,416	83,141	12,681	22,797	38.4/61.6	134	85 (7)	8 (4)	41 (8)	LC160131 [This study]
*M*. *sinensis*	141,372	83,163	12,659	22,775	38.4/61.6	134	85 (7)	8 (4)	41 (8)	NC028721 [33]
*M*. *sacchariflorus*	141,332	83,207	12,575	22,775	38.4/61.6	134	85 (7)	8 (4)	41 (8)	NC028720 [33]
*S*. *bicolor*	140,754	82,688	12,502	22,782	38.5/61.5	135	86 (7)	8 (4)	41 (8)	NC008602 [36]

^1^ Length is indicated in base pairs.

^2^ Including genes detected in this study (not annotated in GenBank).

^3^ Including *ycf*15 and *ycf*68.

^4^ The numbers of duplicated genes are shown in parentheses.

### Sequence variations in cp genomes

We compared sequences determined in this study with those previously registered in GenBank (28 pertial sequences including 10 regions for *E*. *arundinaceus* and the whole cp genome sequence for *M*. *sinensis*). For *E*. *arundinaceus*, sequence variations were identified at ten sites in seven regions, of which four sites (in *trn*G–*trn*fM, *atp*B–*rbc*L, *trn*K intron, and *rpl*16 intron) were mutated in repeat regions (poly A or T). Base substitutions were detected at six sites (*atp*A–*rps*14, three sites in the *rpl*16 intron, *rps*16–*trn*Q, and *rps*3). In the *atp*A–*rps*14 intergenic spacer region we found an adenine-to-cytosine transition (A-to-C; A in Japanese accessions and C in Indonesian accessions), which could reflect geographical variation (S1 Fig). Detailed comparison between the *M*. *sinensis* sequence determined in this study and the previously reported one [33] (NC028721) detected three SNPs and nine indels. Of these, an indel in *rpo*C2 and a SNP in *ycf*3 resulted in amino acid sequence changes (S2 Table).

### Whole-genome comparison in the *Saccharum* complex

**Fig 2:**
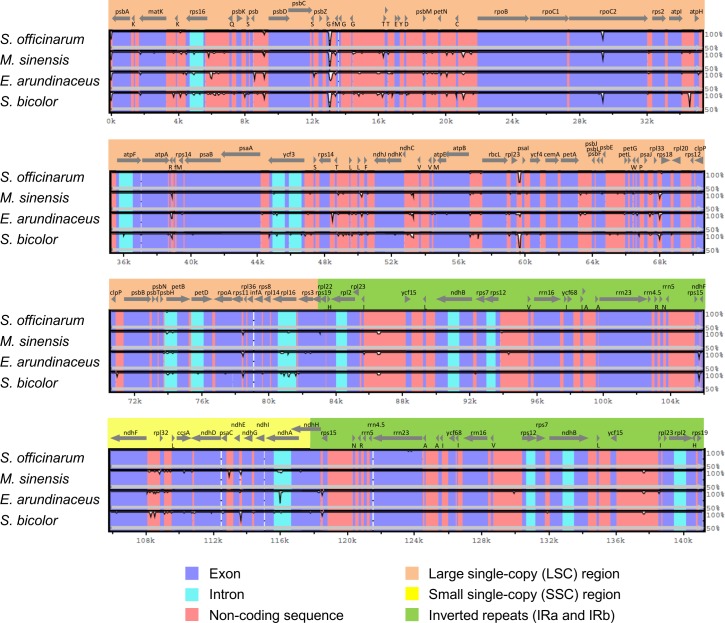
Alignment of whole chloroplast genome sequences from four Panicoideae species. Chloroplast genomes were aligned by using the mVISTA program with the *Zea mays* sequence as a reference. The *X*- and *Y*-scales indicate the coordinates within cp genomes and the percentage of identity (50%–100%), respectively. Genome regions (exons, introns, and conserved non-coding sequences) are color-coded. Gray arrows indicate the direction of transcription of each gene. The genes encoding transfer RNAs (*trn*) are indicated under gray arrows using the single-letter amino acid code (e.g., K: *trn*K).

A global alignment of the *Saccharum* complex cp genomes with the *Zea mays* cp genome (NC001666) as a reference is shown in Fig 2. High sequence similarities in the protein-coding regions were detected. The IR regions showed lower levels of sequence divergence than the single-copy regions, although there was some gene loss. The gene order was identical in *E*. *arundinaceus*, *M*. *sinensis*, and *S*. *officinarum*. However, detailed comparisons within the *Saccharum* complex revealed a number of SNPs and indels (Table 3). The rates of SNP substitutions (nonsynonymous [d*N*] and synonymous [d*S*]) and their ratio (d*N* / d*S*) among the 76 protein-coding genes in comparison with those of *Z*. *mays* are shown in Table 4. The d*N* (0.0039) and d*S* (0.0170) values of *E*. *arundinaceus* were slightly higher than those of the other genera. The d*N*/d*S* values of the *Saccharum* complex were smaller than 1.0, similar to those of other Poaceae [40–42]; these values suggest purifying selection of the cp protein-coding genes in these genera.

**Table 3:** SNPs and indels between *Erianthus arundinaceus* and *Miscanthus sinensis* chloroplast genomes.

Category	SNP	Indel	Total
Photosystem I	*psa*A (2), *psa*C (1)		3
Photosystem II	*psb*B (4), *psb*C (4), *psb*D (1), *psb*E (4), *psb*M (1), *psb*N (1), *psb*T (1), *psb*Z (1)		17
ATP synthase	*atp*A (1), *atp*B (4), *atp*I (2)		7
Cytochrome	*pet*A (2), *pet*B (2), *pet*D (3),	*pet*B (1)	8
NADPH	*ndh*A (1), *ndh*B (1), *ndh*C (2), *ndh*D (6), *ndh*F (7), *ndh*G (3), *ndh*H (6), *ndh*I (1), *ndh*J (3), *ndh*K (2)	*ndh*A (1)	33
Transcription	*rpo*A (4), *rpo*B (11), *rpo*C1 (7), *rpo*C2 (14)		36
Ribosomal proteins (Small subunit)	*rps*2 (1), *rps*3 (4), *rps*8 (1), *rps*11 (1), *rps*14 (2), *rps*15 (1), *rps*18 (2)		12
Ribosomal proteins (Large subunit)	*rpl*14 (1), *rpl*16 (1), *rpl*20 (2), *rpl*22 (1), *rpl*32 (1), *rpl*33 (1)		7
Other	*inf*A (1), *ycf*3 (2), *ycf*4 (1), *ycf*68 (1), *mat*K (12), *ccs*A (5)	*mat*K (1), *ccs*A (1)	24
Rubisco	*rbc*L (5)		5
Non-coding	Intron (38), IGS^1^ (360)	124	522
Total	546 (148) ^2^	128	674

^1^ Intergenic spacer region.

^2^ Parenthesis shows SNPs in protein-coding genes.

**Table 4:** Substitution rates on 76 protein-coding chloroplast genes in three genera of the *Saccharum* complex and *Sorghum bicolor*.

Species	Substitution rate ^1^		d*N* / d*S*
	d*N*	d*S*	
*S*. *officinarum*	0.0030±0.0007	0.0144±0.0030	0.2460
*E*. *arundinaceus*	0.0039±0.0010	0.0170±0.0030	0.2433
*M*. *sinensis*	0.0036±0.0010	0.0152±0.0030	0.2548
*S*. *bicolor*	0.0038±0.0009	0.0183±0.0028	0.2140

^1^ d*N*: the rates of nonsynonymous, d*S*: the rates of synonymous substitutions, *Zea mays* was used as a reference.

The distribution of microsatellites (also called simple sequence repeats) in the cp genomes of *E*. *arundinaceus* and *M*. *sinensis* is shown in Table 5. A total of 40 microsatellite regions (≥8 bp) were identified in *E*. *arundinaceus*, including 36 mono-, 3 tri-, and one tetranucleotide repeats. In *M*. *sinensis*, a total of 38 regions were identified, including 36 mono-, one tri-, and one tetranucleotide repeats. The majority of repeats were located in non-coding regions, whereas some were found in genes such as *psb*C, *rpo*B, *ndh*K, *inf*A, and *rpl*22. Two microsatellites (in *rps*16–*trn*Q/UUG and *trn*R/UCU–*trn*fM/CAU) were found in *E*. *arundinaceus* but not in *M*. *sinensis*.

**Table 5:** Microsatellites in *Erianthus arundinaceus* and *Miscanthus sinensis* chloroplast genomes.

Location	Motif	*E*. *arundinaceus*			*M*. *sinensis*		
		Sequence	Start	End	Sequence	Start	End
*mat*K-*trn*K/UUU	Mono	(A/T)8	3548	3555	(A/T)10	3550	3560
*mat*K-*trn*K/UUU	Mono	(A/T)15	3756	3771	(A/T)13	3760	3773
*trn*K/UUU-*rps*16	Mono	(A/T)11	4118	4129	(A/T)10	4120	4130
*rps*16-*trn*Q/UUG	Tri	(ATT)4	5844	5856	-	-	-
*rps*16-*trn*Q/UUG	Mono	(A/T)11	6417	6428	(A/T)13	6214	6227
*psb*K-*psb*I	Mono	(A/T)10	7757	7767	(A/T)14	6471	6485
*trn*S/GCU-*psb*D	Mono	(A/T)12	9067	9079	(A/T)10	8746	8756
*psb*C	Mono	(G/C)10	11033	11043	(G/C)10	11032	11042
*trn*G/GCC-*trn*fM/CAU	Mono	(A/T)10	13447	13457	(A/T)12	13440	13452
*trn*T/GGU-*trn*E/UUC	Mono	(A/T)15	16614	16629	(A/T)11	16638	16649
*trn*T/GGU-*trn*E/UUC	Mono	(A/T)11	16708	16719	(A/T)9	16728	16736
*trn*D/GUC-*psb*M	Mono	(A/T)14	18717	18731	(A/T)11	18736	18747
*psb*M-*pet*N	Mono	(A/T)14	19267	19281	(A/T)11	19279	19290
*trn*C/GCA-*rpo*B	Mono	(A/T)11	21124	21135	(A/T)13	21111	21124
*rpo*B	Mono	(A/T)10	31970	31980	(A/T)10	31961	31971
*atp*I-*atp*H	Mono	(A/T)10	34148	34158	(A/T)14	34140	34154
*atp*I-*atp*H	Mono	(A/T)9	34684	34692	(A/T)12	34696	34708
*atp*F intron	Mono	(A/T)9	35871	35879	(A/T)10	35892	35902
*atp*A-*trn*R/UCU	Mono	(A/T)9	38743	38751	(A/T)10	38764	38774
*trn*R/UCU-*trn*fM/CAU	Tri	(ATT)7	38901	38922	-	-	-
*psa*A-*ycf*III	Mono	(A/T)13	44325	44338	(A/T)13	45675	45688
*trn*T/UGU-*trn*L/UAA	Mono	(A/T)11	48910	48921	(A/T)8	48896	48903
*trn*L/UAA-*trn*F/GAA	Mono	(A/T)10	50274	50284	(A/T)8	50282	50289
*ndh*K	Mono	(A/T)14	52432	52446	(A/T)14	52438	52452
*trn*M/CAU-*atp*E	Tetra	(AGGT)4	54731	54747	(AGGT)3	54736	54747
*atp*E-*rbc*L	Mono	(A/T)12	56799	56811	(A/T)11	56982	56993
*atp*E-*rbc*L	Mono	(A/T)12	57457	57469	(A/T)11	57454	57465
*rpl*23-*psa*I	Mono	(A/T)10	59774	59784	(A/T)8	59777	59784
*psa*I-*ycf*4	Mono	(A/T)10	60225	60235	(A/T)9	60226	60234
*pet*A-*psb*J	Mono	(A/T)11	63645	63656	(A/T)9	63644	63652
*psb*E-*pet*L	Mono	(A/T)14	65805	65819	(A/T)10	65604	65614
*rpl*33-*rps*18	Mono	(A/T)12	68260	68272	(A/T)12	68219	68231
*pet*B intron	Mono	(A/T)12	74056	74068	(A/T)11	78555	78566
*inf*A	Mono	(A/T)10	79145	79155	(A/T)10	79104	79114
*inf*A	Mono	(A/T)10	79163	79173	(A/T)10	79122	79132
*rpl*16 intron	Mono	(A/T)13	81363	81376	(A/T)15	81328	81343
*rps*3-*rpl*22	Mono	(A/T)10	82624	82634	(A/T)10	81698	81708
*rpl*22	Tri	(CTT)4	83063	83075	(CTT)4	83028	83040
*rpl*32-*trn*L/UAG	Mono	(A/T)9	109365	109373	(A/T)11	109392	109403
*ndh*A intron	Mono	(A/T)7	116146	116152	(A/T)12	116318	116330

### Phylogenetic analyses

**Fig 3:**
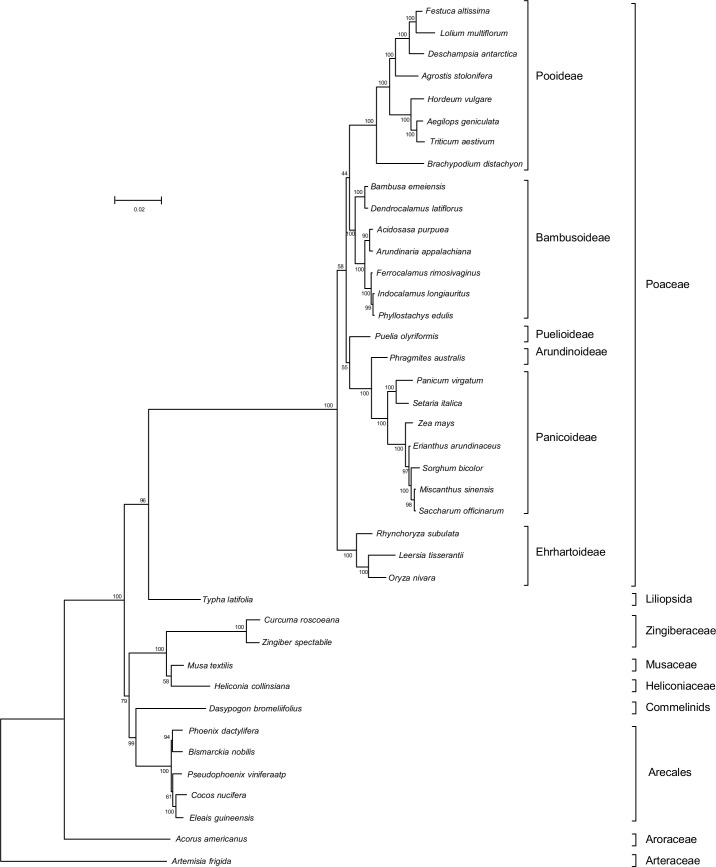
Phylogenetic analysis of 40 species including three genera of the *Saccharum* complex. A phylogenetic tree was generated using the maximum-likelihood method based on the concatenated nucleotide sequences of 76 protein-coding chloroplast genes. Numbers beside the nodes indicate the bootstrap values (%) from 1,000 replicates.

Phylogenetic analyses were performed on an alignment of concatenated nucleotide sequences of 76 protein-coding genes from 40 angiosperm species (39 monocots and one dicot). After all positions containing gaps and missing data were excluded, the final dataset contained a total of 17,396 nucleotide sequences. Maximum likelihood (ML) analysis resulted in a single tree with the highest log-likelihood (lnL) of −89413.4029. Of the 37 nodes, 29 had bootstrap values of ≥95% and 24 of these had bootstrap values of 100% (Fig 3). Maximum parsimony (MP) analysis generated a single most parsimonious tree with a length of 11,454 (consistency index, 0.57; retention index, 0.86; data not shown). The ML and MP trees had similar topology, which was also similar to those of the published phylogenetic trees of grasses based on complete cp genomes [37, 43]. The 39 monocot taxa were divided into two major groups, one containing Poales, including the *Saccharum* complex, and the other one containing all other monocots. *E*. *arundinaceus*, *M*. *sinensis*, and *S*. *officinarum* were grouped into the PACMAD clade, which is one of the major Poaceae lineages. *S*. *officinarum* was more closely related to *M*. *sinensis* than to *E*. *arundinaceus*, in line with previous phylogenetic analyses [14, 44].

### Divergence time estimates

**Fig 4:**
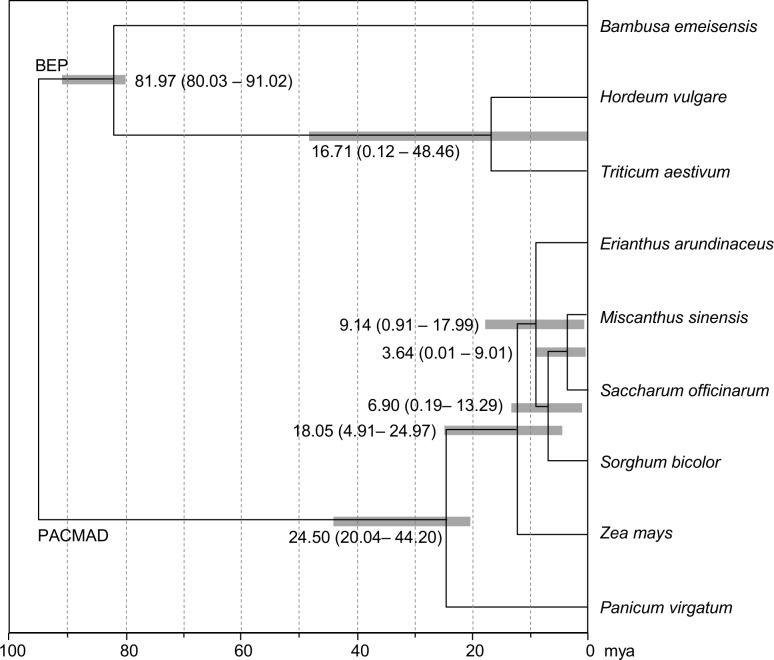
Divergence times of the PACMAD clade. A Bayesian relaxed-clock approach based on 76 concatenated protein-coding chloroplast genes was used to estimate divergence times.

Using 76 concatenated protein-coding genes from the PACMAD clade, including the *Saccharum* complex, we estimated the divergence time with the Bayesian approach assuming a relaxed lognormal clock with the constrained calibration point of the oldest C_4_ lineage in Chloridoideae. As shown in Fig 4, the BEP and the PACMAD clades diverged 81.97 million years ago (mya). Within the PACMAD clade, *Panicum virgatum* (Paniceae) diverged from the other species 24.50 mya (range, 20.04–44.20 mya). *E*. *arundinaceus* was estimated to have diverged from the other genera of the *Saccharum* complex 9.14 mya (range, 0.91–17.99 mya), whereas *M*. *sinensis* and *S*. *officinarum* diverged approximately 3.64 mya (range, 0.01–9.01 mya).

## Discussion

### Features of the chloroplast genomes of *E*. *arundinaceus* and *M*. *sinensis*

In this study, we determined the complete cp genome sequences of the members of the *Saccharum* complex, *E*. *arundinaceus* and *M*. *sinensis*, using 454 GS FLX pyrosequencing and Sanger sequencing. Pyrosequencing has been increasingly used for the sequencing of entire cp genomes, including those of species from several genera of the Poaceae family [33, 36, 37], because of its high throughput and low cost. However, homopolymer stretches (mononucleotide repeats) cause errors in pyrosequencing data; these errors are generally difficult to correct by increasing sequence read depth [45, 46]. In addition, alignment gaps are often allowed in the assembled sequences [45]. In this study, we designed 258 primers, which made it possible to complete sequencing of the entire *E*. *arundinaceus* cp genome, and applied these primers to *M*. *sinensis*. These primers have high identity with other plant cp genome sequences such as those of *S*. *officinarum* and *S*. *bicolor* (S1 Table), and could be used, together with pyrosequencing, for resequencing of ambiguous sites such as homopolymeric and gap regions in Poaceae cp genomes, but also for sequencing of entire cp genomes.

Homopolymers are often present in cp genomes and may be used as microsatellite markers. Because the cp genome sequences are highly conserved among grasses, microsatellite primers for cp genomes are transferable across species and genera. In addition, homopolymers are highly polymorphic, and are valuable markers for the analysis of differentiation and population structure, although overall the cp genome sequences are highly conserved. Inter- and intraspecific variations of cp microsatellites have been used to estimate the genetic diversity and phylogenetic relationships among species and genera [47]. With a threshold of ≥8 bp, we found 40 microsatellite loci for *E*. *arundinaceus* and 38 for *M*. *sinensis*, including 3 tri- and one tetranucleotide repeats, which were located mostly in non-coding regions. This information could be useful for the development of microsatellite markers for the analysis of genetic diversity in *Erianthus*, *Miscanthus*, and related genera.

### Comparison of the sequences within and among *Saccharum* complex species

Comparison of the sequences determined in this study and the sequences previously registered in GenBank identified some polymorphisms. Most of them were found in homopolymeric regions in *E*. *arundinaceus*. A base substitution identified in the *atp*A–*rps*14 intergenic spacer region reflects geographic heterogeneity. Comparison of the whole cp genome sequences of two *M*. *sinensis* accessions detected SNPs and indels at 12 sites. These results indicated the presence of intraspecific mutations in the highly conservative cp genome and could be useful for the analysis of genetic diversity and evolution of *Erianthus*, *Miscanthus*, and related genera. However, Yook *et al*. [48] have reported (on the basis of phenotypic and nuclear SSR genotypic analyses) that some *M*. *sinensis* accessions, including those used for cp genome sequencing, might be hybrids with *M*. *sacchariflorus*. Further studies are required to validate intraspecific mutations in *M*. *sinensis*.

The gene contents differ slightly among the three genera of the *Saccharum* complex because of a frame-shift mutation that resulted in a premature stop codon and loss of the hypothetical gene *ycf*68 in these genera. Similar mutations have been reported in some other plant species [49]. Intact copies of another hypothetical gene, *ycf*15, were detected in both *E*. *arundinaceus* and *M*. *sinensis* cp genomes, although in some other species this gene contains several internal stop codons and is thus nonfunctional [49]. The validity of *ycf*15 and *ycf*68 as protein-coding genes is questionable: according to Raubeson *et al*. [50], their pattern of evolution is not consistent with them encoding proteins. Therefore, these genes were excluded from subsequent analysis in this study and further investigation is required to understand their functions.

### Phylogenetic relationships and evolution

Our phylogenetic analysis based on the variation of the nucleotide sequences of 76 protein-coding genes in cp genomes separated Poales from other monocot groups with a bootstrap value of 100%, which is largely consistent with a recent analysis of other cp genome sequences [40]. Our data suggest that *S*. *officinarum* is more closely related to *M*. *sinensis* than to *E*. *arundinaceus*. We estimated that *S*. *officinarum* and *M*. *sinensis* diverged 3.6 mya, which is in good agreement with divergence times previously estimated on the basis of nuclear (3.1–3.8 mya) genome diversity [51, 52]. A study based on restriction fragment length polymorphism analysis, which used 12 cp-specific probes and examined 32 *Saccharum* complex genotypes, showed that *Erianthus* diverged from other lineages early in the evolution of the subtribe Saccharinae [14]. Our analysis estimated the divergence time as 9.1 mya. In addition, *E*. *arundinaceus* diverged from the subtribe Sorghinae before the divergence of *S*. *bicolor* and the common ancestor of *S*. *officinarum* and *M*. *sinensis*. The present study showed that the cp genome of *E*. *arundinaceus* is more closely related to that of *S*. *bicolor* than to those of other members of the *Saccharum* complex. These data support the suggestion of Sobral *et al*. [14] that the evolutionary history of *Erianthus* may differ from that of other members of the *Saccharum* complex.

In the Old World, *Erianthus* species comprise four cytotypes: diploid (2*n* = 2*x* = 20), triploid (2*n* = 3*x* = 30), tetraploid (2*n* = 4*x* = 40), and hexaploid (2*n* = 6*x* = 60), with a basic number of *x* = 10 [4]. The present study does not clarify how *Erianthus* was established, and additional investigations are required. Inclusion of different cytotypes in phylogenetic analysis based on cp genome sequences may provide useful information on the origin and establishment of this genus. Maternal origin of hybrids and polyploids of several species has been investigated using cpDNA variations [53–55]. The use of combined data on nuclear and cpDNA variations may help determine the origin and evolutionary history of polyploids [56]. In the subtribe Saccharinae, comparative analysis of nuclear genome variations in *Saccharum* and *Miscanthus* suggested that a whole-genome duplication occurred in their common ancestor [51]. This molecular phylogenetic approach, which is used to elucidate the origin and history of polyploidization, could also contribute to characterization of the phylogenetic relationships of *Erianthus*. Therefore, understanding nuclear genome variations, especially in low-copy nuclear loci [52, 57], together with cp genome variations would also be useful for clarifying the evolution of the *Erianthus* polyploid complex. Understanding its evolution could help us to gain more insight into the phylogenetic relationships of the *Saccharum* complex genera and provide useful information on their ancestor and polyploidization, which is critical for genetic studies and breeding in these genera.

## Conclusion

Comparison of the complete cp genomes provided detailed information on genetic variations among three economically important genera, *Saccharum*, *Erianthus*, and *Miscanthus*. Comparison of the sequences indicated that *S*. *officinarum* and *M*. *sinensis* are more closely related to each other than to *E*. *arundinaceus*. We suggest that *E*. *arundinaceus* diverged from the subtribe Sorghinae before the divergence of *S*. *bicolor* and the common ancestor of *S*. *officinarum* and *M*. *sinensis*. This is the first report of phylogenetic and evolutionary relationships among the three genera of the *Saccharum* complex inferred from maternally inherited variations in whole cp genomes and gene data sets. Our results provide an important framework for understanding the phylogeny and evolutionary history of the *Saccharum* complex. Molecular data for the other genera of the complex, *Narenga* and *Sclerostachya*, are limited and further studies on these genera are needed to improve our understanding of the phylogeny and evolution of the *Saccharum* complex.

## Materials and Methods

### Plant materials and DNA extraction

The *E*. *arundinaceus* accession JW630 (Genebank accession number JP173957 at the Genetic Resources Center of the National Institute of Agrobiological Sciences, Japan; https://www.gene.affrc.go.jp/index_en.php) is a wild hexaploid collected in Shizuoka prefecture, Japan (the northernmost area of the wild *E*. *arundinaceus* range in Japan). The *M*. *sinensis* accession Niigata 410 (JP177091) is a wild diploid collected in Niigata prefecture, Japan. Plants were cultivated in a greenhouse at the National Agriculture and Food Research Organization, Institute of Livestock and Grassland Science (NARO-ILGS), and genomic DNA was isolated from fresh green leaves using the CTAB method [58].

### *E*. *arundinaceus* chloroplast genome sequencing and assembly

The *E*. *arundinaceus* cp genome was sequenced by using pyrosequencing. Total *E*. *arundinaceus* genomic DNA was sheared by nebulization and then amplified by emulsion PCR. Amplification products were sequenced on a 454 GS FLX Titanium platform (Roche, Basel, Switzerland) [59]. Chloroplast sequence reads were extracted by local BLASTN searches using the cp genome of *S*. *officinarum* [31] as a reference and assembled with Newbler software (v 2.5; Roche). Homopolymer regions (poly A/T and poly G/C) and the junctions between single-copy regions (LSC and SSC) and IRs were amplified and confirmed using primers designed from the *E*. *arundinaceus* cp sequence (S1 Table) and PrimeSTAR HS DNA polymerase (TaKaRa, Shiga, Japan). PCR products were purified in a QuickStep2 PCR Purification system (Edge Biosystems, Gaithersburg, MD, USA). They were cycle-sequenced with a BigDye Terminator Cycle Sequence Kit v3.1 (Life Technologies, Foster city, CA, USA) and sequenced using an ABI3130xl genetic analyzer (Life Technologies) using primers described below (S1 Table).

### *M*. *sinensis* chloroplast genome sequencing

The *M*. *sinensis* cp genome was sequenced by using Sanger sequencing of PCR products. Sixteen primers to amplify overlapping products (1,747–12,913 bp) were designed from the *E*. *arundinaceus* cp genome sequence for initial amplification of the *M*. *sinensis* cp genome (Table 1). Amplification reactions and cycle-sequencing were performed as described above for *E*. *arundinaceus*. A total of 258 primers (S1 Table) were used to sequence the entire *M*. *sinensis* cp genome.

### Annotation, microsatellite analysis, and comparison of the chloroplast genomes

The entire sequences of the *E*. *arundinaceus* and *M*. *sinensis* cp genomes were annotated using Dual Organellar GenoMe Annotator (DOGMA) software [60]. The predicted annotations were manually checked and verified by comparison with sequences from other PACMAD clade species. The circular chloroplast genome maps were drawn by GenomeVx software [61].

Microsatellites were predicted using MSATCOMMANDER 1.03 software [62]. We defined microsatellites as ≥10 repeats (10 bases) for mononucleotides, ≥8 repeats (16 bases) for dinucleotides, ≥5 repeats (15 bases) for trinucleotides, ≥4 repeats (16 bases) for tetranucleotides, ≥4 repeats (20 bases) for pentanucleotides, and ≥4 repeats (24 bases) for hexanucleotides.

Genome structures among the genera of the *Saccharum* complex were compared using mVISTA software in Shuffle-LAGAN mode [63]; sequence annotation of *Z*. *mays* was used.

### Substitution rates

Substitution rates were calculated using the PAMLX package [64]. The program CODEML in PAMLX was employed to estimate the rates of nonsynonymous (d*N*) and synonymous (d*S*) substitutions and their ratio (d*N* / d*S*) in 76 cp protein-coding genes aligned by using PAL2NAL [65]. The maximum likelihood (ML) tree (see below) was used as a topologically constrained tree. The F3 × 4 model was adopted for codon frequencies under the branch-site model (model = 2, NSsites = 2, and cleandata = 1).

### Phylogenetic analysis

Nucleotide sequences of 76 cp protein-coding genes of 37 monocot angiosperms and one dicot angiosperm (*Artemisia frigida*) available in the GenBank database, and those of *E*. *arundinaceus* and *M*. *sinensis* were concatenated and aligned using Clustal W [66]. After manual editing, phylogenetic analyses using ML and maximum parsimony (MP) were performed with MEGA6 [67] using subtree-pruning-regrafting and nearest-neighbor-interchange algorithms, respectively. The gaps in the alignment were treated as missing data and statistical support at each node was assessed by bootstrapping [68] with 1,000 replicates. Bootstrap values are indicated on the tree.

### Estimation of divergence time of the *Saccharum* complex

A set of 76 protein-coding genes was aligned and used for the estimation of divergence time. The analysis was performed with nine species including three species of the *Saccharum* complex with a focus on the PACMAD clade (Fig 4) using the BEAST2 program, which infers tree topology, branch lengths, and node ages by using Bayesian inference and Markov Chain Monte Carlo (MCMC) analysis [69]. The AIC (Akaike Information Criterion) analysis was performed by using jModelTest 2.1.6 [70] to identify the best fit of the substitution model for mutation rates. BEAUti in the BEAST2 program was used to set the criteria for the analysis. We used the GTR (general-time reversible) model of nucleotide substitution with five categories of gamma-distributed rate. An uncorrelated lognormal model of rate variation among branches was assumed and a Yule prior on the birth rate of new lineages was employed [71]. A single divergence time was previously estimated, assuming that the major diversification of the grass groups occurred 80 mya and the Andropogoneae crown diverged 20 mya [72, 73]; these two time points were used to calibrate the age of the stem nodes. Two independent MCMC runs were performed for 10 million generations with tree sampling every 1,000 generations. The results were checked with Tracer 1.6 [74], and the sampled trees were summarized by using TreeAnnotator v.2.1.2 available in the BEAST2 package, and edited by using FigTree v.1.4.2 [75]. The mean and the estimated 95% highest posterior density interval for the divergence time are given for the major PACMAD lineages.

## Supporting Information

S1 Fig Sequence variations detected among the whole cp genome (sequenced in this study) and partial sequences (registered in Genbank) from *Erianthus arundinaceus*. Origin is indicated as follows: JPN, Japan; IDN, Indonesia; IND, India; THA, Thailand.
(PPTX)

S1 Table Sequencing primers designed from the *Erianthus arundinaceus* cp genome. (XLSX)

S2 Table Summary of sequence variations detected between chloroplast genome sequences of two *Miscanthus sinensis* accessions. (DOCX)
