# Supplementary material for: Complete Chloroplast Genomes of Erianthus arundinaceus and Miscanthus sinensis: Comparative Genomics and Evolution of the Saccharum Complex
Source: PLoS One. 2017 Jan 26;12(1):e0169992. doi: 10.1371/journal.pone.0169992 (PMC5268433; doi:10.1371/journal.pone.0169992)
Supplement: S1 Fig — Origin is indicated as follows: JPN, Japan; IDN, Indonesia; IND, India; THA, Thailand. (PPTX) [file pone.0169992.s001.pptx]

## Slide 1
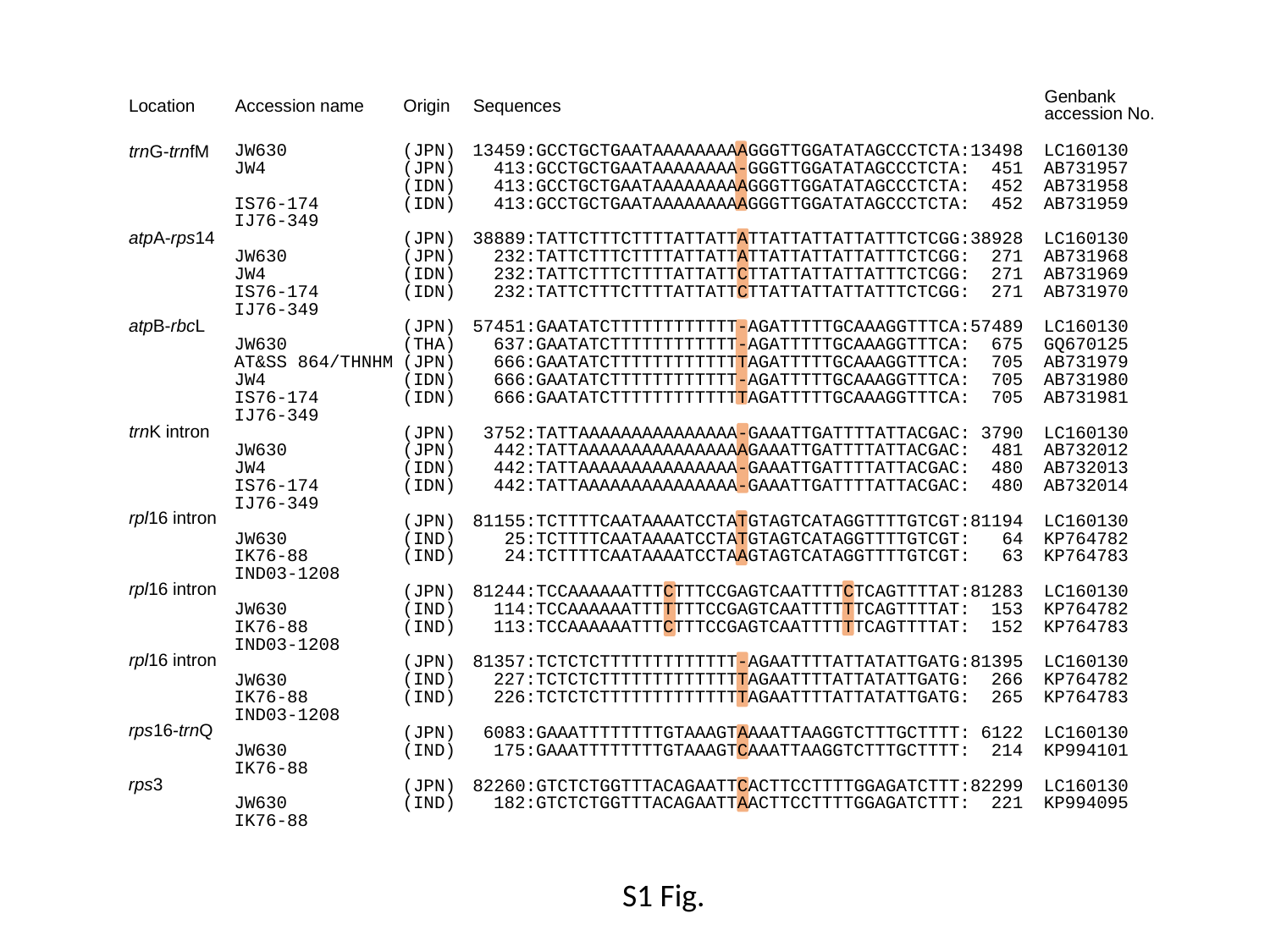

Genbank
accession No.
Location
Accession name
Origin
Sequences
JW630
JW4
IS76-174
IJ76-349
JW630
JW4
IS76-174
IJ76-349
JW630
AT&SS 864/THNHM
JW4
IS76-174
IJ76-349
JW630
JW4
IS76-174
IJ76-349
JW630
IK76-88
IND03-1208
JW630
IK76-88
IND03-1208
JW630
IK76-88
IND03-1208
JW630
IK76-88
JW630
IK76-88
(JPN)
(JPN)
(IDN)
(IDN)
(JPN)
(JPN)
(IDN)
(IDN)
(JPN)
(THA)
(JPN)
(IDN)
(IDN)
(JPN)
(JPN)
(IDN)
(IDN)
(JPN)
(IND)
(IND)
(JPN)
(IND)
(IND)
(JPN)
(IND)
(IND)
(JPN)
(IND)
(JPN)
(IND)
13459:GCCTGCTGAATAAAAAAAAAGGGTTGGATATAGCCCTCTA:13498
 413:GCCTGCTGAATAAAAAAAA-GGGTTGGATATAGCCCTCTA: 451
 413:GCCTGCTGAATAAAAAAAAAGGGTTGGATATAGCCCTCTA: 452
 413:GCCTGCTGAATAAAAAAAAAGGGTTGGATATAGCCCTCTA: 452
38889:TATTCTTTCTTTTATTATTATTATTATTATTATTTCTCGG:38928
 232:TATTCTTTCTTTTATTATTATTATTATTATTATTTCTCGG: 271
 232:TATTCTTTCTTTTATTATTCTTATTATTATTATTTCTCGG: 271
 232:TATTCTTTCTTTTATTATTCTTATTATTATTATTTCTCGG: 271
57451:GAATATCTTTTTTTTTTTT-AGATTTTTGCAAAGGTTTCA:57489
 637:GAATATCTTTTTTTTTTTT-AGATTTTTGCAAAGGTTTCA: 675
 666:GAATATCTTTTTTTTTTTTTAGATTTTTGCAAAGGTTTCA: 705
 666:GAATATCTTTTTTTTTTTT-AGATTTTTGCAAAGGTTTCA: 705
 666:GAATATCTTTTTTTTTTTTTAGATTTTTGCAAAGGTTTCA: 705
 3752:TATTAAAAAAAAAAAAAAA-GAAATTGATTTTATTACGAC: 3790
 442:TATTAAAAAAAAAAAAAAAAGAAATTGATTTTATTACGAC: 481
 442:TATTAAAAAAAAAAAAAAA-GAAATTGATTTTATTACGAC: 480
 442:TATTAAAAAAAAAAAAAAA-GAAATTGATTTTATTACGAC: 480
81155:TCTTTTCAATAAAATCCTATGTAGTCATAGGTTTTGTCGT:81194
 25:TCTTTTCAATAAAATCCTATGTAGTCATAGGTTTTGTCGT: 64
 24:TCTTTTCAATAAAATCCTAAGTAGTCATAGGTTTTGTCGT: 63
81244:TCCAAAAAATTTCTTTCCGAGTCAATTTTCTCAGTTTTAT:81283
 114:TCCAAAAAATTTTTTTCCGAGTCAATTTTTTCAGTTTTAT: 153
 113:TCCAAAAAATTTCTTTCCGAGTCAATTTTTTCAGTTTTAT: 152
81357:TCTCTCTTTTTTTTTTTTT-AGAATTTTATTATATTGATG:81395
 227:TCTCTCTTTTTTTTTTTTTTAGAATTTTATTATATTGATG: 266
 226:TCTCTCTTTTTTTTTTTTTTAGAATTTTATTATATTGATG: 265
 6083:GAAATTTTTTTTGTAAAGTAAAATTAAGGTCTTTGCTTTT: 6122
 175:GAAATTTTTTTTGTAAAGTCAAATTAAGGTCTTTGCTTTT: 214
82260:GTCTCTGGTTTACAGAATTCACTTCCTTTTGGAGATCTTT:82299
 182:GTCTCTGGTTTACAGAATTAACTTCCTTTTGGAGATCTTT: 221
LC160130
AB731957
AB731958
AB731959
LC160130
AB731968
AB731969
AB731970
LC160130
GQ670125
AB731979
AB731980
AB731981
LC160130
AB732012
AB732013
AB732014
LC160130
KP764782
KP764783
LC160130
KP764782
KP764783
LC160130
KP764782
KP764783
LC160130
KP994101
LC160130
KP994095
trnG-trnfM
atpA-rps14
atpB-rbcL
trnK intron
rpl16 intron
rpl16 intron
rpl16 intron
rps16-trnQ
rps3
S1 Fig.
