# Supplementary material for: Complete Chloroplast Genomes of Erianthus arundinaceus and Miscanthus sinensis: Comparative Genomics and Evolution of the Saccharum Complex
Source: PLoS One. 2017 Jan 26;12(1):e0169992. doi: 10.1371/journal.pone.0169992 (PMC5268433; doi:10.1371/journal.pone.0169992)
Supplement: S2 Table — (DOCX) [file pone.0169992.s003.docx]

**S2 Table Summary of polymorphisms detected between chloroplast genome sequences of two *Miscanthus sinensis* accessions**

| **Polymorphism** | **Location** | **Nucleotide sequence^1^** | **Amino acid sequence** |
| --- | --- | --- | --- |
| SNP | *rps*16–*trn*Q | 6194:CCTAAAAGAAAGAAAGAACT**A**AAAAAAAAAAAATAAGAAAA: 6234  6194:CCTAAAAGAAAGAAAGAACT**C**AAAAAAAAAAAATAAGAAAA: 6234 | - |
| Indel | *psb*K–*psb*I | 8007:GCAATTCTTGCAATTTTTTT**T**AAGAATTGATTTTTGCATTT: 8046  8007:GCAATTCTTGCAATTTTTTT-AAGAATTGATTTTTGCATTT: 8046 | - |
| Indel | *trn*G–*trn*T | 16345:TACATATATTAT**ATATATACATATATTAT**GTACATTAATAT: 16385  16344:TACATATATTAT-----------------GTACATTAATAT: 16367 | - |
| SNP | *trn*C–*rpo*B | 21354:CTGATATAGATTATAGCCTG**G**CCTTCTTGGCCCACCTAAGT: 21394  21336:CTGATATAGATTATAGCCTG**C**CCTTCTTGGCCCACCTAAGT: 21376 | - |
| Indel | *rpo*C2 | 29536:TATAGGACCCGAGAGGG**AGAGGG**CGAATATGAAATCCTAGA: 29576  29518:TATAGGACCCGAGAGGG------CGAATATGAAATCCTAGA: 29552 | 683:RTREG**EG**EYEIL:694  683:RTREG--EYEIL:692 |
| Indel | *atp*F intron | 35882:ATTTTTAGTATTTTTTTTTT-ATAAATAAGAAAAGGTGCAC: 35921  35858:ATTTTTAGTATTTTTTTTTT**T**ATAAATAAGAAAAGGTGCAC: 35898 | - |
| Indel | *ycf*3 intron | 45667:CTCCTTTCTTTTTTTTTTTT**T**CTTTCTATAGTGGAGATAGT: 45707  45644:CTCCTTTCTTTTTTTTTTTT-CTTTCTATAGTGGAGATAGT: 45683 | - |
| SNP | *ycf*3 | 45800:TGTATGCTCTCCATTGCTTG**G**GTGTATAAGGCCTATGTTAT: 45840  45776:TGTATGCTCTCCATTGCTTG**T**GTGTATAAGGCCTATGTTAT: 45816 | 78:IGLIH**P**SNGEHT: 89  78:IGLIH**T**SNGEHT: 89 |
| Indel | *atp*B–*rbc*L | 56767:ACATAATTTTCAAAAAAAAA-GGAATTTGTCGAAATTTTTT: 56806  56743:ACATAATTTTCAAAAAAAAA**A**GGAATTTGTCGAAATTTTTT: 56783 | - |
| Indel | *rpl*33–*rps*18 | 68210:AAAGAAATCTTTTTTTTTTT**T**CAAATTTTTAAATAAGGAAT: 68250  68187:AAAGAAATCTTTTTTTTTTT-CAAATTTTTAAATAAGGAAT: 68226 | - |
| Indel | *ndh*E–*ndh*G | 113849:GAAGGTATTT**AAAAGAGAAAAGAAGGTATTT**GTTGTGTTGG:113889  113825:GAAGGTATTT---------------------GTTGTGTTGG:113844 | - |
| Indel | *ndh*A intron | 116310:GTATACTTAAAAAAAAAAAA-GAATAAAGGGTTAATTCGTT:116349  116265:GTATACTTAAAAAAAAAAAA**A**GAATAAAGGGTTAATTCGTT:116305 | - |

^1^ The upper and lower parts indicate *M. sinensis* chloroplast genome sequences from LC160131 (in this study) and NC028721 [33], respectively. The SNPs and indels are marked with bold capitals.
